# Quality of Patient-Provider Communication Among Cancer Survivors: Findings From a Nationally Representative Sample

**DOI:** 10.1200/JOP.2015.006999

**Published:** 2016-05-24

**Authors:** Neetu Chawla, Danielle Blanch-Hartigan, Katherine S. Virgo, Donatus U. Ekwueme, Xuesong Han, Laura Forsythe, Juan Rodriguez, Timothy S. McNeel, K. Robin Yabroff

**Affiliations:** Kaiser Permanente Northern California, Oakland, CA; National Cancer Institute, Bethesda; and Information Management Services, Rockville, MD; Bentley University, Waltham, MA; Rollins School of Public Health, Emory University; Centers for Disease Control and Prevention; and American Cancer Society, Atlanta, GA; and Patient-Centered Outcomes Research Institute, Washington, DC

## Abstract

**Purpose:**

Although patient-provider communication is an essential component of health care delivery, little is known about the quality of these discussions among patients with cancer.

**Methods:**

Data are from the 2011 Medical Expenditure Panel Survey Experiences with Cancer survey among 1,202 adult cancer survivors. We evaluated discussions with any provider after a cancer diagnosis about: (1) follow-up care; (2) late or long-term treatment effects; (3) lifestyle recommendations, such as diet, exercise, and quitting smoking; and (4) emotional or social needs. Using a response scale ranging from “did not discuss” to “discussed in detail,” a summary score was constructed to define communication quality as high, medium, or low. Patient factors associated with the quality of provider discussions were examined using multivariable polytomous logistic regression analyses.

**Results:**

At the time of the survey, approximately one half of the patients (46%) were either within 1 year (24.1%) or between 1 and 5 years (22.0%) of treatment. More than one third of cancer survivors reported that they did not receive detailed communication about follow-up care, and more than one half reported that they did not receive detailed communication regarding late or long-term effects, lifestyle recommendations, or emotional and social needs. Only 24% reported high-quality communication for all four elements, indicating that the vast majority experienced suboptimal communication. In multivariable analysis, survivors reporting a high communication quality with providers included those who were within 1 year of treatment, between the ages of 18 and 64 years, non-Hispanic black or other ethnicity, and married.

**Conclusion:**

Study findings demonstrate gaps in the communication quality experienced by cancer survivors in the United States and help identify survivors for targeted interventions.

## INTRODUCTION

In 2014, approximately 14.5 million individuals in the United States had a history of a cancer diagnosis, and this population is expected to grow because of advancements in cancer detection and treatment and the aging of the population.^1-4^ Patient-provider communication is central to the care experiences of cancer survivors, who face many challenging decisions relating to the management of late and long-term treatment effects, surveillance for recurrence or new primaries, and psychosocial needs.^5-11^

Survivorship care planning has been highlighted as one way to assist providers in communicating with patients with cancer and reducing gaps in care by promoting detailed discussions of patient needs, addressing quality-of-life concerns, providing cancer-related information, and outlining strategies to meet ongoing health needs.^7,12-16^ In the 2006 Institute of Medicine report “From Cancer Patient to Cancer Survivor: Lost in Transition,” and the 2011 LIVESTRONG Foundation consensus statement, national experts asserted that survivorship care planning should incorporate certain essential elements, including information about surveillance for recurrence, adverse effects of treatment, psychosocial needs, and lifestyle recommendations, to adequately address survivorship needs.^7,16^

Existing research on survivorship care planning has examined the prevalence of care plans and has highlighted the need for increased use.^17-20^ These studies have found that only a third of patients report receiving survivorship care plans,^17^ less than 5% of oncologists report consistently providing and discussing care plans with their patients,^18^ and less than half of National Cancer Institute–designated comprehensive cancer centers provide care plans to patients.^19^Furthermore, available measures of patient-provider communication among cancer survivors typically address whether discussions occurred about specific topics or whether treatment summaries were provided. However, little is known about the quality or depth of discussions between cancer survivors and their providers regarding survivorship care topics.

In this study, we used nationally representative data from the Medical Expenditure Panel Survey (MEPS) Experiences with Cancer survey to examine: (1) the quality of communication regarding the key elements of survivorship care planning, including follow-up care recommendations; late or long-term adverse effects of cancer treatment; support for emotional or social needs; and lifestyle recommendations such as diet, exercise, and quitting smoking; and (2) patient factors associated with the quality of discussions with providers. Our findings will help identify gaps in the quality of communication between cancer survivors and their health care providers in the United States and will help identify survivors who could benefit from targeted interventions to improve patient-provider communication.

## METHODS

### Data Source and Sample

The MEPS is an ongoing, nationally representative survey of the civilian noninstitutionalized population of the United States.^21^ The MEPS Experiences with Cancer survey was a self-administered questionnaire distributed to adult cancer survivors identified from a question about whether a physician or other health professional had ever told the person that he/she had cancer or a malignancy of any kind.^22^ In 2011, cancer survivors who had received a cancer diagnosis and/or cancer treatment after age 18 years were eligible to complete the survey, which contained questions on financial burden, access to medical care, employment, and health care use related to cancer. The MEPS response rate was 54.9% overall, and among MEPS participants, the Experiences with Cancer survey response rate was 90%, resulting in a final response rate of 49.4%. In total, we identified 1,202 adult cancer survivors for this study. We excluded individuals diagnosed solely with nonmelanoma skin cancer, as is typical in other cancer survivorship studies.^23,24^

### Measures

#### Patient-Provider Communication During Survivorship Care

The outcome assessed was patient-provider communication during survivorship care, as measured by the question, “At any time since you were first diagnosed with cancer, did any doctor or other healthcare provider, including your current healthcare provider, ever discuss with you – (1) The need for regular follow-up care and monitoring even after completing your treatment?; (2) Late or long-term side effects of cancer treatment you may experience over time?; (3) Your emotional or social needs related to your cancer, its treatment, or the lasting effects of that treatment?; and (4) Lifestyle or health recommendations such as diet, exercise, quitting smoking?” Possible responses for each of these four content areas were: “discussed it with me in detail,” “briefly discussed it with me,” “did not discuss it at all,” or “I don’t remember.”

Response options were scored in the following manner: “discussed in detail” (2), “briefly discussed” (1), “did not discuss at all” (0), “I don’t remember” (0), and missing (0). As illustrated in Appendix Table A1 (online only), a summary score was created by adding up scores from the four questions, resulting in a three-level outcome variable to assess the quality of communication with providers: high (7 and 8), medium (4, 5, and 6), and low (0, 1, 2, and 3). The categorization was developed on the basis of the distribution of the summary score and by drawing on existing literature.

High communication quality was defined as at least three “discussed in detail” responses and no “did not discuss”/“I don’t remember”/missing responses for any of the four items. Low-quality communication was defined as one or more “did not discuss”/“I don’t remember”/missing responses and one or no “discussed in detail” responses. Medium communication quality was defined by multiple combinations of “discussed in detail,” “briefly discussed,” and “did not discuss”/“I don’t remember”/missing responses.

#### Sample Characteristics

Sample characteristics included age (18 to 49 years; 50 to 64 years; 65 to 74 years; ≥ 75 years), sex, race/ethnicity (non-Hispanic white, non-Hispanic black, Hispanic/other/multiple), marital status (married, not married), educational attainment (≤ high school graduate, ≥ some college), general health status (excellent/very good, good, fair/poor), number of provider visits in the previous 12 months (fewer than three, three or more). Time since last cancer treatment was measured in four categories (< 1 year, 1 to < 5 years, 5 to < 10 years, ≥ 10 years) or as never treated/missing.

### Data Analyses

We calculated descriptive statistics for cancer survivors and each of the key measures of communication quality. We also assessed communication quality by time since last cancer treatment and conducted polytomous multivariable logistic regression analyses to examine patient factors associated with communication quality. We present information for high-, medium-, and low-quality communication but focus our discussion of results on predictors of optimal communication (ie, high), consistent with other literature examining patient experiences with care, because patient reports of communication with providers are typically skewed toward positive care experiences.^25-28^

Patient factors were selected for inclusion in our final regression models on the basis of existing literature and bivariate associations with communication quality. In several cases, we chose one of several related factors. For example, given the high proportion of insured individuals in the sample (96%), we chose the number of annual provider visits instead of health insurance coverage. We present adjusted predicted margins, which directly standardize the outcome of each group to the covariate distribution of the overall population.^29^ All estimates were weighted to account for the MEPS complex survey design and survey nonresponse using SUDAAN.^30^ Wald statistics were used to test the statistical significance of covariates in multivariable analyses. All tests of statistical significance were two sided.

Because respondents who said they did not know whether providers discussed a topic with them could be considered as having poor communication, we coded responses of “I don’t remember/missing” (n = 297) from our outcome measure the same way as respondents saying “did not discuss at all” (ie, scored as 0). This decision was based on the rationale that respondents who said they did not know whether providers discussed a topic with them would not be able to follow recommendations to take action, such as making lifestyle modifications to reduce the risk of recurrence or to pursue recommended follow-up care. However, we conducted a sensitivity analysis excluding these respondents to assess whether the pattern of results differed from our main analyses. Because results were similar, we present findings from the full sample, where “I don’t remember/missing” was considered poor communication. We also conducted additional sensitivity analyses including insurance coverage and cancer site. The results were consistent with those of our main analyses and are not presented because of sample size constraints.

## RESULTS

Sample characteristics are presented in Table 1. The majority of survivors were 65 years old or older (54.6%), female (57.5%), non-Hispanic white (85.9%), and married (57.2%), and reported some college education or more (57.0%). Nearly half (42.6%) reported excellent or very good health status, and approximately three quarters (80.9%) reported having three or more annual provider visits. Similar proportions were in each category for time since treatment, with approximately one quarter reporting that they were within a year of treatment (24.1%).

**Table 1.**
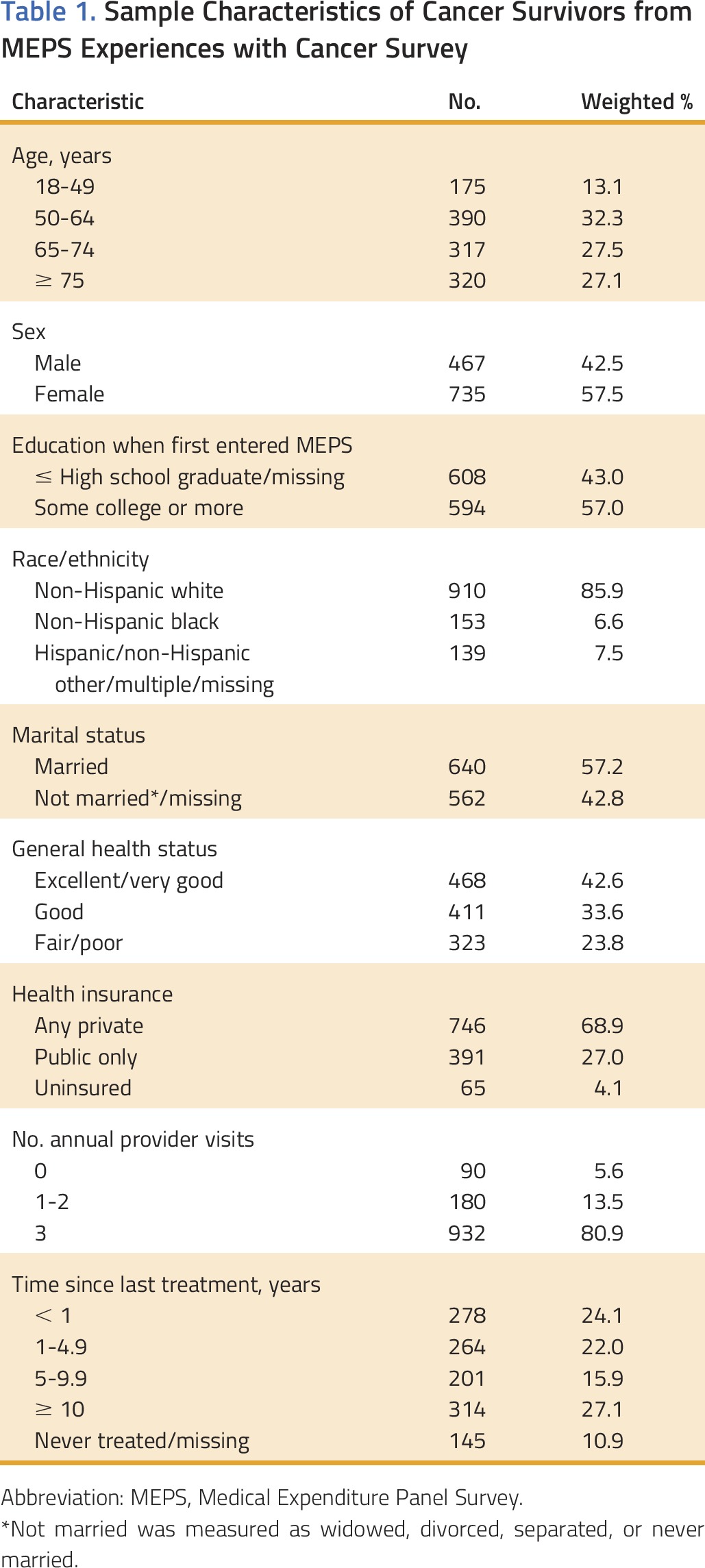
Sample Characteristics of Cancer Survivors from MEPS Experiences with Cancer Survey

**FIG 1.**
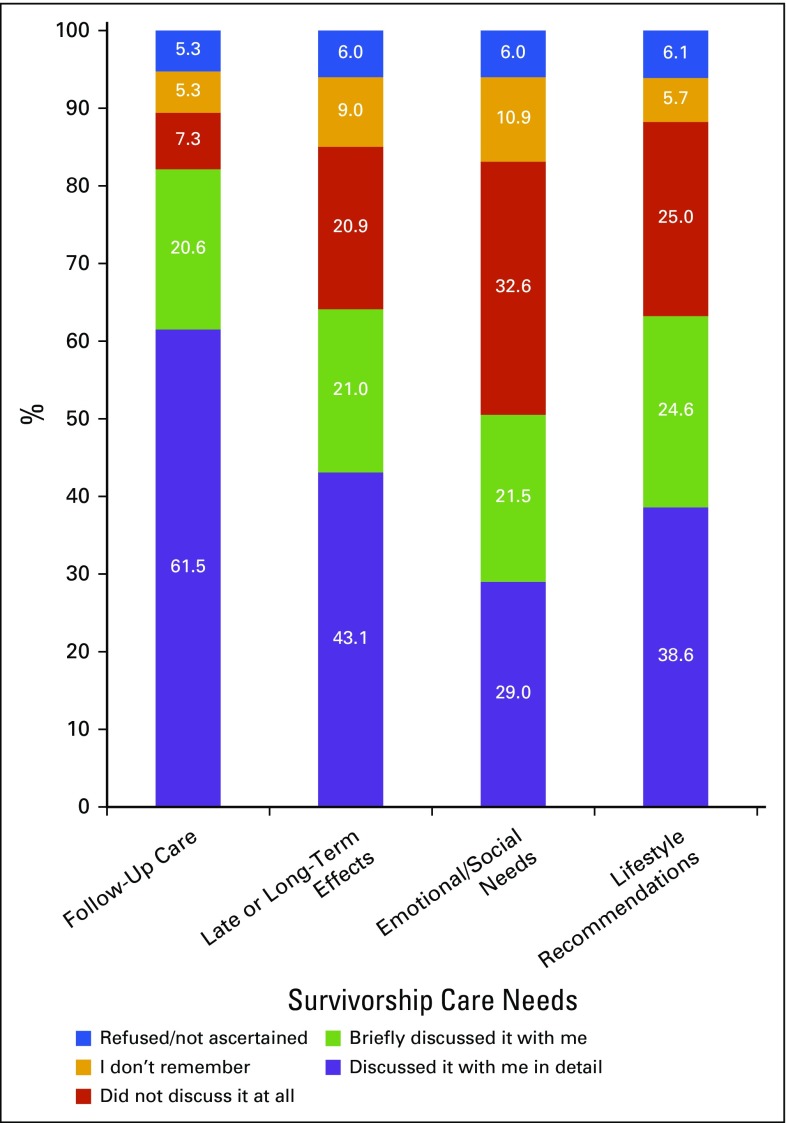
Communication quality between cancer survivors and their providers regarding survivorship care needs (unadjusted).

Figure 1 depicts unadjusted proportions of cancer survivors reporting their communication quality with providers after their cancer diagnosis regarding the four survivorship content areas assessed. Approximately one third reported that they did not receive detailed communication about follow-up care, and more than half did not receive detailed communication regarding late or long-term effects, emotional or social needs, or lifestyle recommendations. The proportions who received no communication were sizable and varied by content area, ranging from 7.3% for follow-up care to 32.6% for emotional needs. In addition, only one of four cancer survivors (24.4%) reported that the physician discussed all four of these content areas in detail (data not shown).

We also examined communication quality by time since last treatment (data not shown). Detailed communication was significantly lower for patients further from their last treatment, compared with more recently treated patients, regarding the need for regular follow-up care and monitoring (*P*_trend_ = .022), the long-term adverse effects of cancer treatment (*P*_trend_ < .001), lifestyle or health recommendations (*P*_trend_ = .012), and emotional or social needs (*P*_trend_ = .049).

Adjusted analysis of patient factors associated with high, medium, and low communication quality among survivors is presented in Table 2. Survivors who were more likely to report high-quality communication included those who were within 1 year of treatment versus those who were 10 or more years from diagnosis, 18 to 49 years of age or 50 to 64 years of age versus 75 years of age and older, non-Hispanic black or other ethnicity versus non-Hispanic white, and married versus not married. By contrast, individuals who were never treated/missing, 75 years of age or older, non-Hispanic white, or not married were more likely to report low-quality communication. The associations between sex and educational attainment and communication quality had a nonlinear pattern. For instance, those with at least some college education were more likely to report medium communication quality, and those with less education were more likely to report either high- or low-quality communication with providers.

**Table 2.**
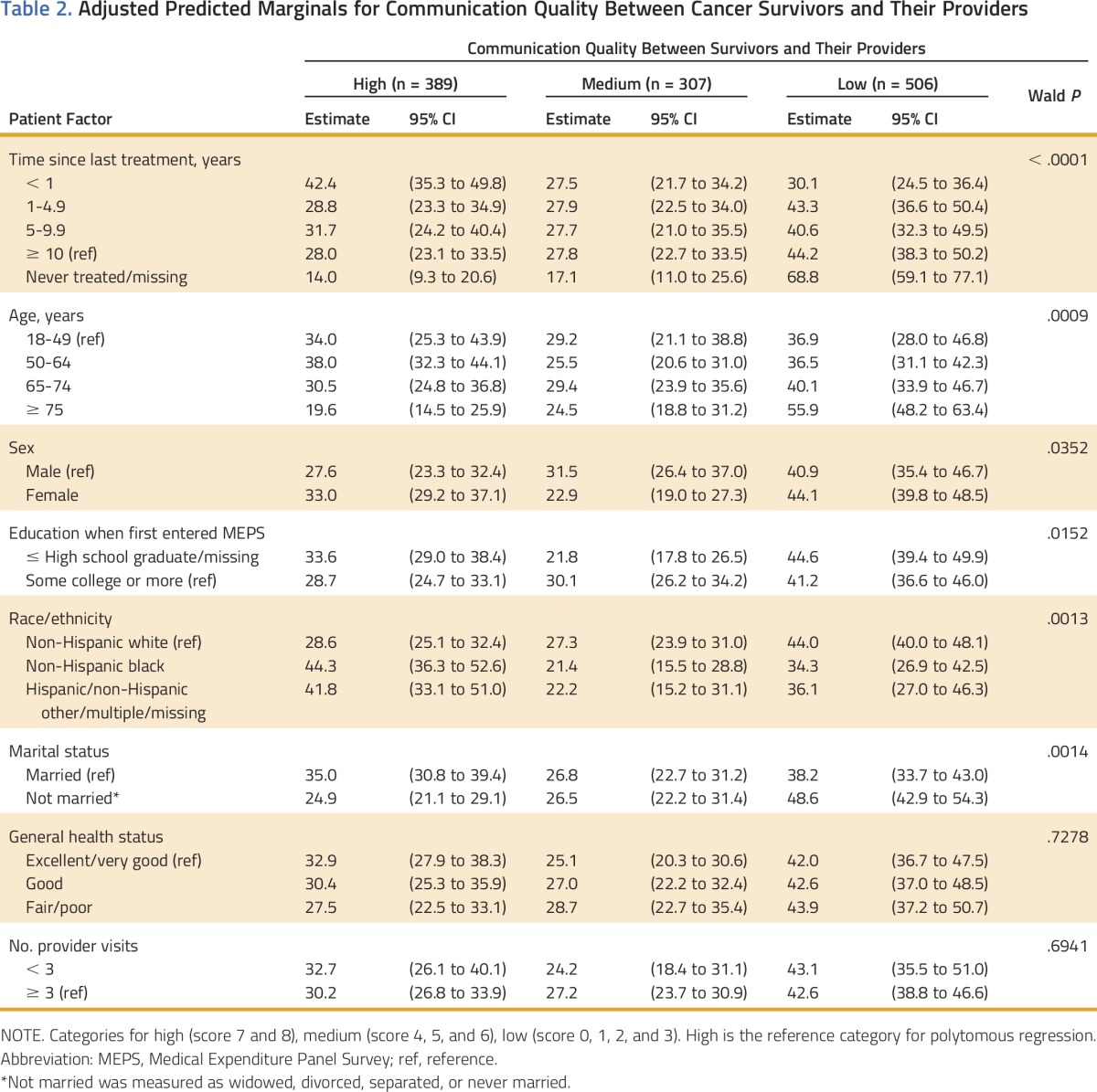
Adjusted Predicted Marginals for Communication Quality Between Cancer Survivors and Their Providers

## DISCUSSION

In this population-based, nationally representative sample, we found that limited proportions of cancer survivors reported high-quality discussions with providers after diagnosis, ranging from 29% for emotional and social needs to 62% for follow-up care recommendations. In addition, only 24% reported high-quality communication for all four elements, indicating that 76% experienced suboptimal communication with their cancer care providers. As underscored by the National Cancer Institute and the Institutes of Medicine, patient-centered communication with cancer care providers is an essential component of delivering high-quality cancer care.^5-7^

Several existing national efforts address aspects of survivorship care planning within the context of cancer care delivery and may ultimately improve the quality of communication. For example, the Commission on Cancer recently mandated the use of survivorship care plans in Commission on Cancer–accredited facilities, where > 70% of newly diagnosed patients are treated, and included survivorship care plans as part of the process for Oncology Medical Home certification.^31^ In late 2014, the Centers for Medicare and Medicaid Services announced that the Medicare Program will reimburse separately for care coordination of patients with multiple chronic conditions, including cancer.^32^ This policy requires physicians to do several things that overlap with key elements of survivorship care planning, such as developing a comprehensive plan for patient care; assessing patients' medical, psychological, and social needs; and monitoring care given by other physicians. The Center for Medicare and Medicaid Innovation has also developed the Oncology Care Model, which is a new payment model that aims to provide higher quality, more highly coordinated cancer care at a lower cost to Medicare patients.^33^ Collectively, these greater efforts may yield improvements in survivorship care planning and communication between providers to enable better coordination of cancer care.^31,32^ Furthermore, ongoing evaluation of the quality of patient-provider discussions for cancer survivors will be important as these policies are implemented, and this study provides initial insights in this area. More research is needed in the areas of organizational, provider, and additional patient-level barriers to having quality discussions with cancer care providers.

Our finding that only 62% of cancer survivors reported that any provider had detailed discussions with them regarding follow-up care highlights a crucial gap in communication. Even more striking was the lack of discussion around the late and long-term effects of treatment. Multiple studies have shown that survivors face many challenging physical and psychological effects of treatment that fundamentally shape their quality of life.^5,7-10^ For only 43% of patients to report that adverse effects of treatment were discussed in detail underlies a clear need for improvement in communication with providers.

Fewer than one third of cancer survivors reported that any of their providers discussed their emotional and social needs in detail (29%); these discussions are particularly vital for cancer survivors, who may experience a range of emotions when transitioning from patient to survivor, including fear of recurrence, uncertainty about future plans, and adjustment to the new normal after cancer.^6,34-36^ In addition, only 39% of the survivors in our sample reported that any provider ever discussed lifestyle recommendations. For many patients, experiencing a cancer diagnosis represents a teachable moment that can inform their future behaviors, and many are eager to make adjustments to diet, exercise, and tobacco use because these represent behaviors within their control that can potentially lower their risk of developing future cancers.^37-39^ Existing studies have shown that, for these reasons, provider recommendations during survivorship care may be more likely to have an impact on improving preventive behaviors.^37-39^

Our findings provide valuable information from the patient perspective and are similar to the findings of previous studies demonstrating suboptimal communication about survivorship care planning from the provider perspective. Nationally representative surveys of oncologists and primary care physicians found that only 32% of oncologists and 12% of primary care physicians reported always discussing recommendations for survivorship care with their patients.^17^ Similarly, Salz et al^19^ found that only 23 of the 53 National Cancer Institute–designated cancer centers (43%) reported using survivorship care plans for their breast or colorectal cancer survivors. However, these studies did not address detailed content or the quality of discussions during survivorship care.

In this study, we also assessed patient factors associated with the quality of patient-provider discussions. We found that survivors who were further away from treatment were less likely to report detailed discussions for all four areas, compared with those who were more recently treated. Although it is possible that survivorship care has improved in more recent years, other studies suggest that longer-term survivors experience communication barriers and feel lost in transition as they move from oncology care back to primary care or other subspecialty care.^7,40-44^ Cancer survivors who were never treated for cancer, such as men engaged in active surveillance for prostate cancer, were more likely to report low-quality communication with their providers. Others have reported that these men may be more likely to perceive worse care compared with those undergoing treatment, although on the basis of patient and physician characteristics, the findings are mixed.^45-47^

Contrary to some existing studies, we found that non-Hispanic white patients were more likely to report poor communication with their providers, compared with patients of non-Hispanic black or other ethnicity.^48-52^ One possibility is that white patients may have higher expectations of communication with their cancer care providers compared with other patients and are, therefore, more critical in their ratings of care. However, it is difficult to interpret this result given the limited sample of minority patients in this study. Future studies should incorporate larger samples of minority patients, including individuals with language barriers, to appropriately evaluate their communication experiences with providers regarding cancer survivorship topics.

Notably, this study had some important limitations. We could not control for cancer site because of the limited sample size, and there may be differences in patient-provider communication among patients with different types of cancer or multiple cancers. Our data did not capture cancer stage and therefore we could not control for cancer severity, although our analyses did adjust for health status. Our study does not address differences in communication by provider type or specialty. Data were self-reported and cross-sectional, limiting our ability to draw causal inferences. Our sample was predominantly composed of non-Hispanic whites. Other studies suggest that minority patients may have different experiences in communicating with their providers than do whites.^48-52^ Lastly, there is the potential for recall bias, particularly for respondents further from treatment. For instance, available literature on patients with cancer suggests that significant differences may exist between physician and patient recall of topics discussed.^47,53^ Furthermore, it is challenging to disentangle reasons behind variations in communication by time since last treatment because of multiple factors changing over time, such as the prevalence of particular cancers, provider training, or actual care delivered to patients with cancer.

Despite these limitations, this study makes several important contributions to the existing literature. First, the measure used to assess communication allowed us to both report on quality of discussions and assess four key elements of survivorship care planning from the perspective of patients with cancer. Second, data from this study allowed us to examine the patient factors associated with high-quality discussions between patients and providers and to identify those experiencing suboptimal communication. Third, data were from a population-based, nationally representative sample of cancer survivors in the United States.

In conclusion, the findings from this work demonstrate gaps in the communication quality experienced by cancer survivors in the United States; many patients, including long-term survivors further from their initial treatment, reported experiencing suboptimal communication with their cancer care providers. Similar to the MEPS Experiences with Cancer survey, future assessments of survivorship care discussions should measure communication quality, not just the presence or absence of survivorship care plans, and examine communication quality within longitudinal cohorts. Further work could examine provider communication with patients diagnosed with different types of cancer and by cancer stage. Additional research is also needed in diverse racial/ethnic, linguistic, and health literacy populations to more fully assess the barriers to high-quality communication between cancer survivors and their health care providers. Finally, future research could examine the role of provider characteristics, including provider type, in the quality of patient-provider communication among cancer survivors.

## Figures and Tables

**Table A1.**
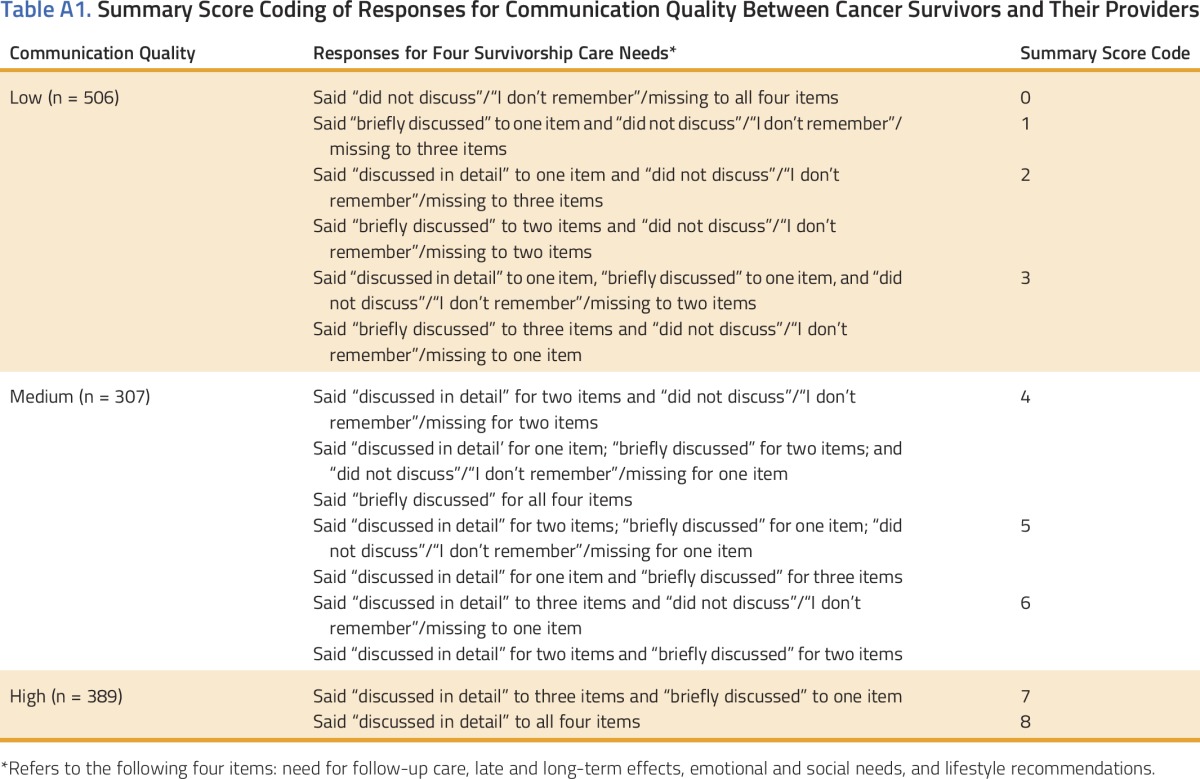
Summary Score Coding of Responses for Communication Quality Between Cancer Survivors and Their Providers
